# Integrated Molecular and Physiological Mechanisms Underlying Rice Adaptation to Heat, Salinity, and Drought Stress

**DOI:** 10.3390/plants15203231

**Published:** 2026-10-21

**Authors:** Atika Khan, Md. Atik Mas-ud, Shahab Ahmad, Shafi Ullah, Mohammad Nurul Matin

**Affiliations:** 1 College of Plant Science and Technology, Huazhong Agricultural University, Wuhan 430070, China; atkakhan7@gmail.com (A.K.); atikgeb@gmail.com (M.A.M.-u.); 2 Department of Agronomy, The University of Agriculture Peshawar, Amir Muhammad Khan Campus, Mardan 02323, Pakistan; shahabahmad3641@gmail.com; 3 Molecular Genetics Laboratory, Department of Genetic Engineering and Biotechnology, University of Rajshahi, Rajshahi 6205, Bangladesh; 4 Department of Biotechnology, Yeungnam University, Gyeongsan 38541, Republic of Korea

**Keywords:** rice, heat stress, salinity stress, drought stress, signal transduction

## Abstract

Rice faces increasing threats from climate change, driven by heat, salinity, and drought stresses. Although researchers have extensively studied individual stresses, growing evidence indicates that combined stresses elicit unique physiological, biochemical, and molecular responses that single-stress studies cannot predict. This review comprehensively synthesizes current knowledge of the integrated signaling networks and adaptive mechanisms underlying rice responses to heat, salinity, and drought stress, with particular emphasis on stress crosstalk and multi-stress responses. We examine the roles of mechanosensitive ion channels, calcium signaling, salt overly sensitive (SOS) pathway, heat shock response regulators, mitogen-activated protein kinase (MAPK) cascades, and ABA-dependent and -independent signaling networks in stress tolerance systems. Furthermore, we discuss major adaptive responses involving reactive oxygen species (ROS) homeostasis, osmoprotectant accumulation, antioxidant defense systems, and phytohormone crosstalk involving abscisic acid, auxin, salicylic acid, cytokinin, brassinosteroids, ethylene, and gibberellins. Particular attention is given to the detrimental effects of combined stresses on growth, development, and yield. In addition, recent advances in enhancing stress tolerance through CRISPR/Cas9-mediated genome editing, marker-assisted breeding, and molecular marker technologies are also highlighted. Despite significant progress, critical knowledge gaps remain regarding primary stress-sensing mechanisms, epigenetic stress memory, organelle-specific signaling, and the translation of laboratory findings to field conditions. By integrating current knowledge across molecular, physiological, and breeding perspectives, we provide a comprehensive integrated signaling network model and crosstalk among signaling pathways for understanding rice adaptation to combined stresses and highlight potential strategies for enhancing crop resilience and global food security.

## 1. Introduction

According to current forecasts, climate change is expected to intensify several environmental challenges, including increased average temperatures, unequal distribution of precipitation in both space and time, intensification of heat-wave frequency, prolonged drought events, and progressive soil salinization, especially in arid and semi-arid regions. These changes pose significant threats to global agricultural productivity and food security. In the economies of many countries of the world, agriculture remains a cornerstone of economic growth and livelihood security. However, the effects of reduced rainfall, increased cases of drought, and the turnover effects of water shortage pose a threat to agriculture. On the other hand, agricultural systems require a large amount of fresh water, which adds to the current water resource limitations [1].

The simultaneous exposure of major crops to abiotic stresses is associated with a substantial decreased of profitability, and these issues represent a concern for global food security [2]. Rice is particularly vulnerable to drought, heat, and salinity, although the magnitude and timing of their effects vary among production environments, cultivars, stress intensity and duration, and developmental stages. These stresses can adversely affect germination, vegetative growth, photosynthesis, reproductive development, grain quality, and final yield, while their simultaneous occurrence may generate physiological and molecular responses that differ from those induced by individual stresses [3].

For example, combined stresses of heat and drought can have negative effects on photosynthesis through reduced stomatal conductance, carbon assimilation, and cellular homeostasis, leading to a decrease in the yield of rice [4]. Likewise, with increased salt absorption, the translocation of ions in the outer cell layer is significantly disrupted due to excessive sodium accumulation, thereby impairing growth and metabolic functions [5]. Although considerable progress has been made in understanding rice responses to individual stresses, knowledge of the adaptive signaling mechanisms governing rice responses to combined heat, salinity, and drought stresses remains limited. It has been revealed that both the stresses of salinity and drought occasioned heavy losses in growth, chlorophyll synthesis, and photosynthesis in many plants [6]. Therefore, creating climate-resilient rice cultivars that can maintain productivity under increasingly difficult climatic conditions requires a thorough understanding of the interrelated molecular, physiological, and biochemical mechanisms involved in multi-stress adaptation. Examining the signaling pathways that detect stress and organize downstream adaptation responses is crucial to comprehending how rice survives in various stress situations.

Rice serves as the primary dietary staple for more than half of the world’s population and therefore plays a central role in global food security [7]. Throughout its life cycle, rice is frequently exposed to various abiotic stresses, among which heat, drought, and salinity are the most destructive. These stresses adversely affect germination, vegetative growth, reproductive development, grain quality, and final yield, and severe stress conditions may even lead to plant mortality [8]. Projections indicate that water scarcity will become a serious constraint for approximately 15–20 million hectares of rice-growing areas, particularly in tropical and low-latitude regions, which may face significant water shortages by 2025 [9]. Moreover, climate change is expected to intensify the frequency and severity of heat waves, drought episodes, and soil salinization, further threatening rice productivity and the livelihoods of millions of smallholder farmers who depend on rice cultivation for their food and income security [10,11].

Given these challenges, developing climate-resilient rice cultivars that can sustain productivity in the face of multiple abiotic stresses has become a top research focus in light of these difficulties. However, the majority of earlier research has concentrated on specific stressors, but rice plants frequently experience simultaneous exposure to heat, salinity, and drought in both natural and cultivated settings. With a focus on integrated signaling networks, stress crosstalk, and adaptive mechanisms that can be used to improve stress tolerance, grain quality, and yield stability under changing climatic conditions, this review attempts to summarize current knowledge on the physiological, biochemical, and molecular responses of rice to these intertwined stresses.

## 2. Methods for Literature Collection

This review followed a structured literature survey to identify peer-reviewed studies addressing molecular, physiological, and hormonal mechanisms underlying rice adaptation to heat, salinity, and drought stress, as well as genome editing and breeding strategies for stress tolerance improvement. Relevant literature was retrieved from Web of Science, Scopus, PubMed, and Google Scholar databases. The search covered publications from 2000 to 2026, with the final search conducted in March 2026. Particular emphasis was placed on recent advances in stress signaling networks, hormone crosstalk, combined-stress responses, and CRISPR/Cas-based approaches published during the last 10 years.

Search queries included combinations of keywords such as heat stress, salinity stress, drought stress, combined abiotic stress, rice, *Oryza sativa*, phytohormones, abscisic acid, auxin, brassinosteroids, gibberellins, jasmonic acid, salicylic acid, ethylene, cytokinins, strigolactones, hormone crosstalk, calcium signaling, ROS signaling, MAPK cascades, transcription factors, HSFs, DREBs, NACs, WRKYs, bZIPs, MYBs, SOS pathway, ion homeostasis, osmotic adjustment, antioxidant defense, genome editing, CRISPR/Cas9, molecular breeding, marker-assisted selection, genomic selection, multi-omics, and crop improvement in conjunction with “rice” or “plants.”

Articles were selected based on the following criteria: (i) original research or authoritative reviews published in English in peer-reviewed journals; (ii) studies reporting molecular, genetic, physiological, or biochemical mechanisms of heat, salinity, and drought stress responses in rice or relevant model plant systems; (iii) research addressing individual and combined stress responses, with emphasis on synergistic interactions among multiple abiotic stresses; (iv) studies providing mechanistic insights into hormone-mediated stress signaling, Ca^2+^ signaling, ROS signaling, MAPK cascades, and transcriptional regulation; and (v) research relevant to crops or model plant systems with translational significance for rice improvement. Studies focusing exclusively on non-target abiotic stresses (e.g., cold, flooding, heavy metals) or lacking mechanistic insight and relevance to heat, salinity, and drought stress adaptation were excluded. Preprints, conference abstracts, and other non-peer-reviewed sources were excluded. Priority was given to original research articles and high-quality reviews.

Papers included in tables and figures were chosen based on their relevance to integrated stress signaling networks, experimental robustness, and contribution to identifying actionable genetic targets for combined stress tolerance in rice. Final synthesis emphasizes consistency across studies and integrates evidence to highlight regulatory nodes, crosstalk mechanisms, and genome editing opportunities for developing climate-resilient rice cultivars. For the combined-stress synthesis, studies were further classified according to stress combination (heat + drought, drought + salinity, heat + salinity, or three-stress exposure), mode of exposure (simultaneous or sequential), stress duration (acute or chronic), developmental stage (vegetative or reproductive), and interaction outcome. Interaction outcomes were interpreted as additive when the combined response approximated the cumulative effects of the component stresses, synergistic when the combined damage or response exceeded that expected from the individual stresses, and antagonistic when one stress partially attenuated the response to another. Single-stress studies were retained only where they provided mechanistic information necessary to interpret experimentally documented combined-stress responses.

## 3. Climate Change and Food Security

Climate change has emerged as one of the greatest threats to global food security, with profound implications for agricultural productivity and the sustainability of cropping systems. Rising temperatures, altered precipitation patterns, prolonged droughts, increasing soil salinity, and the growing frequency of extreme weather events are disrupting crop production worldwide, particularly in developing countries where agriculture remains highly dependent on climatic conditions [12,13]. Rice is especially vulnerable to these environmental changes because of its high sensitivity to water availability and temperature fluctuations. Recent climate projections indicate that climate change will intensify heat waves, exacerbate water scarcity, accelerate soil salinization, and increase the frequency of floods, collectively threatening rice production across major cultivation regions. It is estimated that by 2030, 122 million individuals, who are mainly rural farmers in developing countries, will experience extreme poverty because of climate risks like heavy storms, floods, hurricanes, and drought [14,15]. South and Southeast Asia, which account for the majority of global rice production and consumption, are expected to experience particularly severe impacts owing to increasing climatic variability and the greater occurrence of compound abiotic stresses. Unlike individual stresses, the simultaneous occurrence of heat, drought, and salinity often produces synergistic effects that substantially reduce photosynthetic efficiency, reproductive success, grain filling, and final yield.

In addition to reducing crop productivity, elevated atmospheric CO_2_ and increasing temperatures can adversely affect grain nutritional quality by decreasing the concentrations of protein and essential micronutrients, thereby posing additional challenges to global nutritional security [16]. These combined effects highlight the urgent need to develop climate-resilient rice cultivars capable of maintaining stable yield and grain quality under increasingly complex environmental conditions. Technological developments in genome editing, genomics-assisted selection, and molecular breeding present encouraging prospects to increase rice’s resistance to various abiotic challenges. However, a thorough grasp of the integrated signaling networks and adaptation mechanisms that allow rice plants to recognize, react to, and withstand combined heat, salt, and drought stresses is necessary for the successful implementation of these strategies. Accelerating the creation of robust rice varieties that can support sustainable food production under future climate scenarios requires this kind of research.

## 4. Core Sensing and Signaling Modules Relevant to Combined Abiotic Stress

The following subsections summarize individual stress-sensing components only where they establish mechanistic modules required to interpret combined-stress responses. These pathways should not be viewed as completely independent response systems because they contain shared regulatory components that may converge during combined stress. However, the presence of the same component in multiple single-stress pathways does not demonstrate direct crosstalk, and such interactions are considered established only when supported by combined-stress experiments.

### 4.1. Salt Stress Perception and Signal Transduction

**Figure 1:**
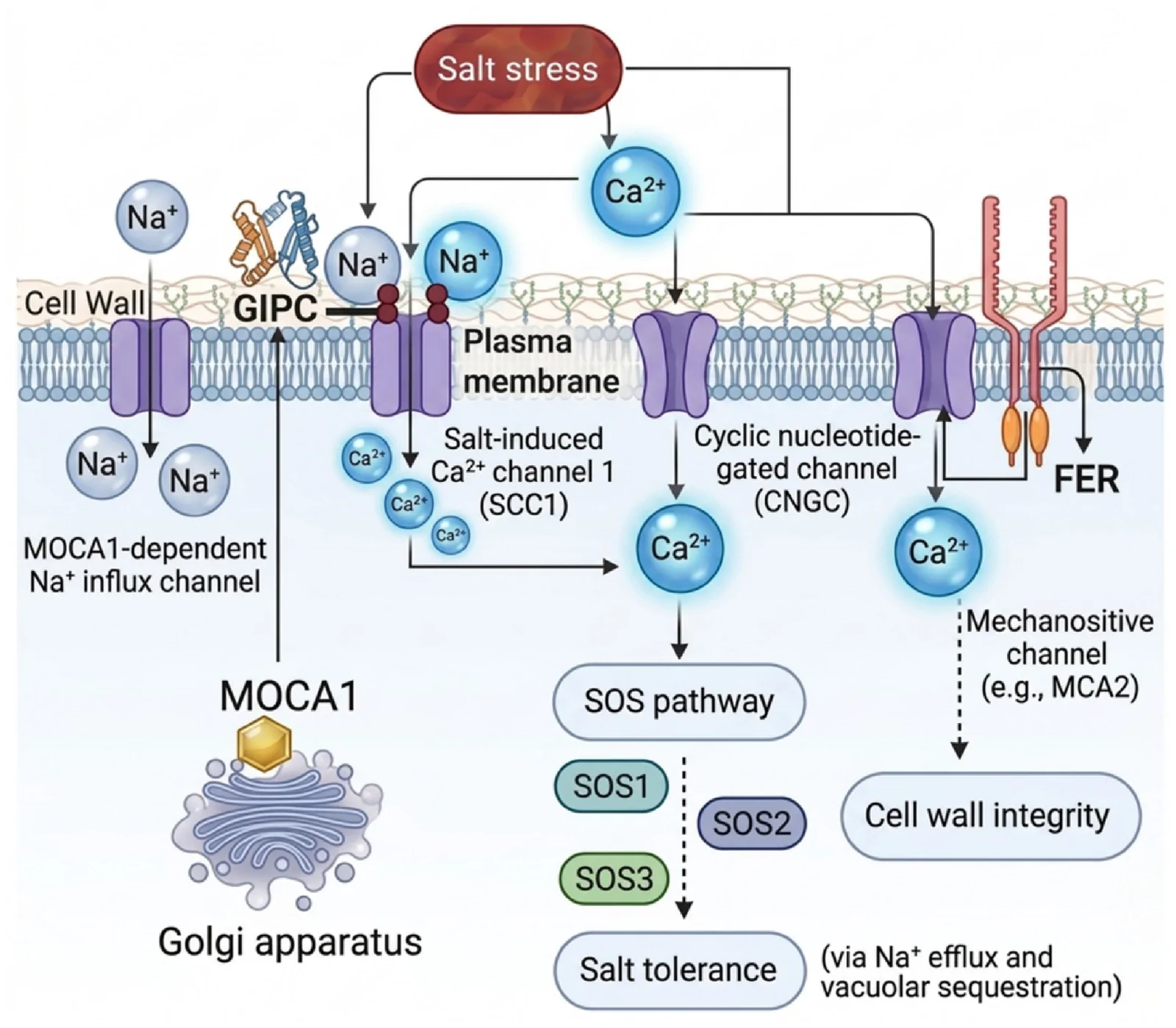
Proposed framework for extracellular salt sensing and early signaling at the cell wall–plasma membrane interface. Salt stress (Na^+^ excess) is associated with changes in glycosylinositol phosphorylceramide (GIPC) sphingolipids, whose biosynthesis requires the glucuronosyltransferase MOCA1. Na^+^ binding to GIPCs is proposed to activate a downstream Ca^2+^-permeable channel, although the identity and precise placement of this channel remain unresolved. This signaling event contributes to the rapid elevation of cytosolic Ca^2+^, potentially involving salt-induced Ca^2+^ channel 1 (SCC1). Cyclic nucleotide-gated channels (CNGCs) may also contribute to salt-induced Ca^2+^ influx and Ca^2+^ transients, which activate the SOS signaling pathway. In this pathway, the SOS3–SOS2 regulatory complex activates the plasma-membrane Na^+^/H^+^ antiporter SOS1, promoting Na^+^ extrusion from the cytosol. In contrast, vacuolar Na^+^ sequestration is mediated by distinct transport systems, including NHX-type Na^+^/H^+^ antiporters, and is therefore not conflated with SOS1-mediated plasma-membrane Na^+^ extrusion. Mechanosensitive channels such as MCA2 and receptor-like kinases such as FERONIA (FER) may contribute to the perception and integration of mechanical or cell-wall-related changes associated with salinity, but they are not considered direct salt sensors without specific supporting evidence. The overall salt-sensing framework and selected signaling relationships were redrawn and adapted from the model presented in the previous study [27]. Pathway organization, transport directions, and terminology were updated based on the primary literature cited in Section 4.1. The figure represents an integrated conceptual model and distinguishes experimentally supported mechanisms from proposed or unresolved components. Created in BioRender. NURUL, M. (2026) https://BioRender.com/n8rzejm.

Salt stress imposes both osmotic and ionic challenges that rapidly disrupt cellular homeostasis. The initial perception of salinity occurs through changes in membrane tension, cell wall integrity, and osmotic potential, which activate a series of interconnected signaling pathways that coordinate adaptive responses. Among the earliest cellular events following salt exposure is a rapid and transient increase in cytosolic calcium (Ca^2+^), which serves as a universal second messenger for transmitting stress signals to downstream regulatory networks [17]. This Ca^2+^ signature is generated within seconds of osmotic perturbation and initiates multiple signaling cascades involved in stress adaptation (Figure 1).

Mechanosensitive ion channels constitute the first line of stress perception by altering mechanical stimuli connected with osmotic imbalance into biochemical signals. The Mechanosensitive Channel of Small Conductance-Like (MSL) family works as non-selective ion channels activated by membrane tension and contributes to organellar osmoregulation, root development, pollen hydration, and protection against cell swelling under hypo-osmotic conditions [18]. Similarly, MID1-Complementing Activity (MCA) proteins are plasma membrane-localized Ca^2+^-permeable channels that respond to mechanical stimuli and participate in root mechanosensing and hypo-osmotic stress responses [19]. Another important mechanosensitive channel, PIEZO1 (PZO1), mediates mechanotransduction during root growth and regulates Ca^2+^ influx in response to mechanical forces. In addition, PIEZO homologs contribute to vacuolar Ca^2+^ homeostasis, polarized cell growth, and pollen tube development, highlighting their roles in plant mechanoperception [20].

Among the identified osmosensors, Reduced Hyperosmolality-Induced [Ca^2+^] Increase 1 (OSCA1) functions as a mechanosensitive Ca^2+^-permeable channel that detects hyperosmotic stress and generates characteristic cytosolic Ca^2+^ signatures essential for osmotic stress tolerance. Recent studies further demonstrate that members of the OSCA protein family operate as stretch-activated ion channels, providing a direct molecular link between membrane deformation and Ca^2+^-mediated signal transduction [21,22]. Likewise, BONZAI (BON) proteins, which are Ca^2+^-dependent phospholipid-binding proteins, positively regulate osmotic stress tolerance by promoting cytosolic Ca^2+^ accumulation, abscisic acid (ABA) biosynthesis, and the expression of stress-responsive genes. BON proteins also interact with plasma membrane Ca^2+^ transport systems to fine-tune early Ca^2+^ signaling during osmotic stress [18,23]. Nevertheless, genetic analyses of *osca* septuple mutants and *bon1 bon2 bon3* triple mutants indicate that additional, yet unidentified, osmotic sensors participate in stress perception, suggesting that plants employ multiple parallel mechanisms to detect environmental perturbations [24].

Calcium sensor proteins, such as calcineurin B-like proteins (CBLs), CBL-interacting protein kinases (CIPKs), calcium-dependent protein kinases (CDPKs), and calmodulins (CaMs), decode the brief increase in cytosolic Ca^2+^ and convert Ca^2+^ signatures into particular downstream reactions. The primary regulatory module governing ionic homeostasis under salt stress is the Salt Overly Sensitive (SOS) signaling pathway. SOS3 (CBL4) detects elevated cytosolic Ca^2+^, which triggers the protein kinase SOS2 (CIPK24). The plasma membrane Na^+^/H^+^ antiporter SOS1 is subsequently phosphorylated and activated by the active SOS2–SOS3 complex, which promotes Na^+^ extrusion from the cytoplasm and maintains intracellular K^+^/Na^+^ equilibrium, which is necessary for cellular survival in saline circumstances. The salt SOS pathway mediates this process, and the mechanism is illustrated in Figure 1 [25].

Salt stress also induces cell wall remodeling, which is monitored by the receptor-like kinase FERONIA (FER). FERONIA senses alterations in cell wall integrity and contributes to salt tolerance by regulating Ca^2+^ influx and maintaining plasma membrane–cell wall adhesion during osmotic stress. Furthermore, FERONIA integrates calcium signaling with ABA signaling through its interaction with the protein phosphatase ABI2, thereby linking osmotic sensing to hormone-mediated stress responses [26]. FERONIA-mediated sensing has been mechanistically established mainly in Arabidopsis; therefore, its corresponding role in rice should presently be regarded as a conserved or putative mechanism unless supported by direct functional evidence in Oryza sativa. Beyond ABA signaling, salt-induced Ca^2+^ signals also interact extensively with ROS, mitogen-activated protein kinase (MAPK) cascades, and stress-responsive transcription factors, including members of the DREB, NAC, WRKY, bZIP, and MYB families. The coordinated activation of these interconnected signaling networks ultimately regulates antioxidant defense, ion transport, osmotic adjustment, cellular protection, and stress-responsive gene expression, enabling plants to maintain growth and enhance tolerance under saline environments.

Collectively, mechanosensitive channels, calcium signaling, the SOS pathway, hormone regulation, ROS signaling, and transcriptional control all work together rather than separately to regulate salt stress perception. Developing rice cultivars with improved resistance to salt and other co-occurring abiotic stressors will require an understanding of the dynamic interactions among these mechanisms.

### 4.2. Temperature Sensing and Signal Transduction

**Figure 2:**
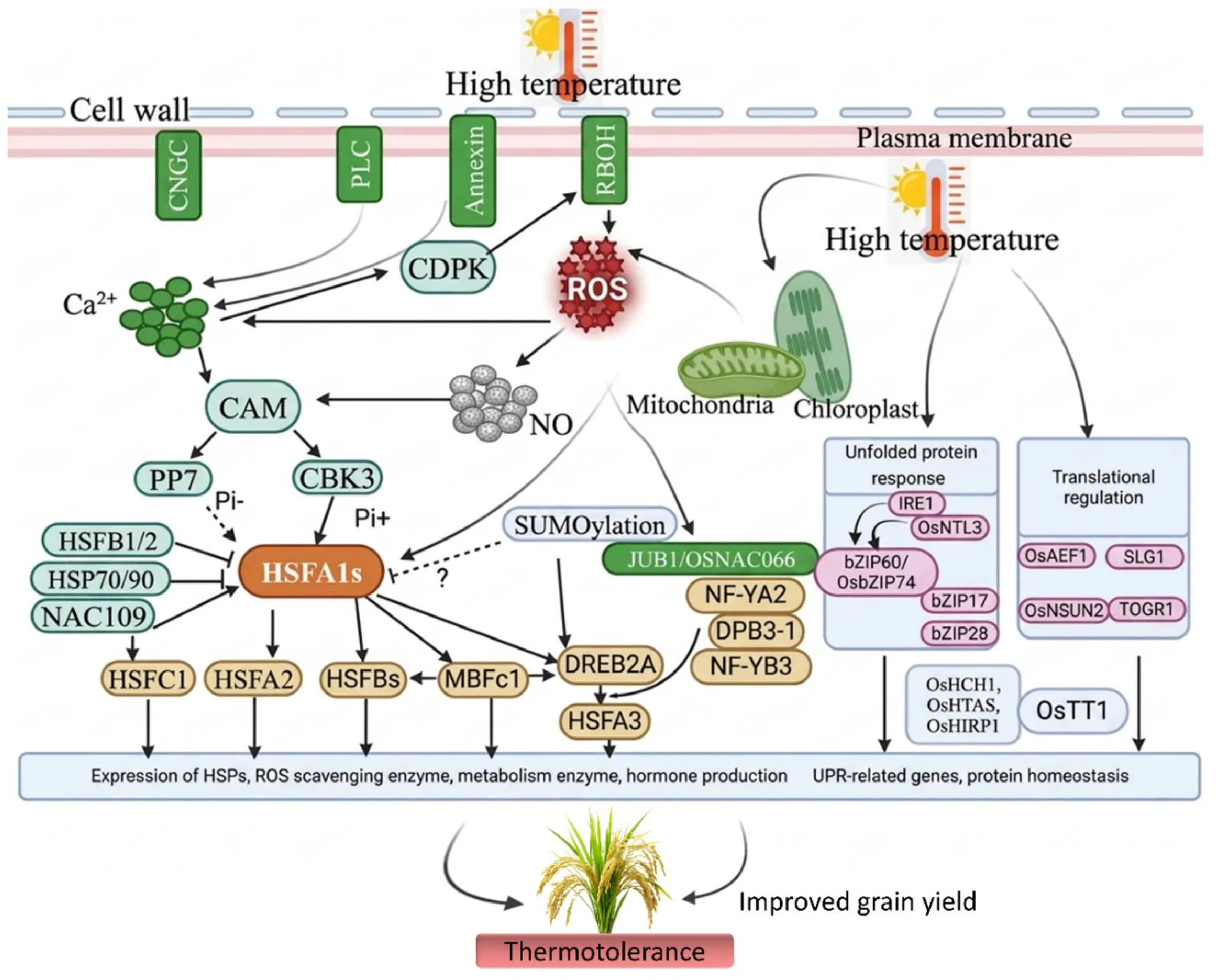
Molecular mechanisms and signaling networks associated with heat stress tolerance in rice. High-temperature stress activates plasma membrane-associated sensors and signaling components, including cyclic nucleotide-gated channels (CNGCs), phospholipase C (PLC), Annexin, and respiratory burst oxidase homologs (RBOH), resulting in cytosolic Ca^2+^ influx. Elevated Ca^2+^ is sensed by calcium-dependent protein kinases (CDPKs) and calmodulin (CAM), which regulate downstream phosphorylation pathways involving PP7 and CBK3 to modulate HSFA1s. Simultaneously, ROS and nitric oxide (NO) generated through RBOH activity act as secondary messengers to amplify heat signaling. Activated HSFA1s coordinate a complex transcriptional network by interacting with HSP70/90, HSBFs, NAC109, DREB2A, HSFA2, HSFC1, MBFc1, and NF-Y complexes, while SUMOylation further fine-tunes transcriptional regulation. Heat stress also disrupts protein homeostasis in the endoplasmic reticulum, chloroplasts, and mitochondria, triggering unfolded protein response (UPR) pathways mediated by IRE1, OsNTL3, bZIP60/OsbZIP74, bZIP17, and bZIP28. In parallel, translational regulators and molecular chaperones, including OsAET1, SLG1, OsNSUN2, TOGR1, OsHCI1, OsHTAS, OsHIRP1, and OsTT1, maintain protein stability and cellular integrity. The coordinated action of these signaling, transcriptional, and organellar pathways ultimately induces HSPs, ROS-scavenging enzymes, metabolic regulators, and stress-responsive genes, thereby establishing thermotolerance in rice. Solid lines indicate mechanisms directly supported in rice, whereas dashed lines indicate relationships inferred primarily from *Arabidopsis* or other species. The core heat signaling framework and selected regulatory relationships were redrawn and adapted from the original model of the recent study [33], while rice-specific components, gene nomenclature, cellular localization, and pathway relationships were updated according to the primary literature cited in Section 4.2. Created in BioRender. NURUL, M. (2026) https://BioRender.com/qjewlrj.

Temperature responses should be distinguished according to their biological context. Thermomorphogenesis refers to temperature-dependent developmental adjustments, whereas basal thermotolerance describes the inherent capacity of plants to withstand heat stress. Acquired thermotolerance develops following prior exposure to a mild heat treatment, whereas heat injury results from excessive or prolonged temperatures that cause membrane destabilization, protein denaturation or aggregation, oxidative damage, impaired photosynthesis, and reproductive dysfunction. These responses are not necessarily governed by the same sensing and signaling mechanisms (Figure 2). The expression of genes necessary for thermotolerance is coordinated by a complex transcriptional network based on Heat Shock Factors (HSFs), which are triggered by these early signaling events.

The HSR is primarily regulated by HSFs, which function as master transcriptional regulators of heat-responsive gene expression. In rice, 25 HSF genes have been identified and classified into three subfamilies: HSFA, HSFB, and HSFC, of which at least 22 exhibit heat-inducible expression [28]. Among these, HSFA1 proteins serve as the principal regulators of the heat-responsive transcriptional network by activating numerous downstream genes involved in cellular protection and stress adaptation [29]. Studies in Arabidopsis have demonstrated that triple mutants lacking *HSFA1a*, *HSFA1b*, and *HSFA1d* exhibit severely impaired induction of heat-responsive genes, including HSFA2, DREB2A, Multiprotein Bridging Factor 1C (MBF1c), HSFBs, and multiple Heat Shock Protein (HSP) genes, highlighting the central regulatory role of HSFA1 proteins during heat stress [30]. Furthermore, HSFA1 directly activates HSFA2, a secondary transcription factor that reinforces and prolongs the heat shock response during sustained thermal stress [31].

It has been demonstrated that a number of rice-specific HSFs support thermotolerance via unique regulatory pathways. Alternative splicing of *OsHSFA2d* is induced by heat stress, indicating that post-transcriptional regulation plays a part in optimizing thermal adaptation [32]. Similarly, *OsHSFA2c* works with *OsHSFB4b* to stimulate transcription of HSP100, a crucial molecular chaperone involved in protein refolding and recovery from heat-induced protein denaturation, while *OsHSFA2e* overexpression increases thermotolerance by activating multiple *HSP* genes [33,34,35]. The significant regulatory roles of class B HSFs during heat acclimation are further supported by the substantial induction of *OsHSFB2b* by high temperatures [36].

Extensive connections between HSFs and other stress-responsive transcription factors reinforce heat-induced transcriptional regulation. By directly activating HSFA3 expression, DREB2A, which was first identified for its function in drought stress responses, also serves as a crucial regulator of thermotolerance [37]. In Arabidopsis, DREB2A promotes HSFA3 transcription and increases the expression of heat-responsive genes by forming a transcriptional complex with Nuclear Factor Y (NF-YA2/NF-YB3) and DNA Polymerase II Subunit B3-1 (DPB3-1) [38]. Loss-of-function mutants of DREB2A, HSFA3, or DPB3-1 exhibit enhanced heat sensitivity, underscoring the importance of this regulatory module [39]. Similar regulatory interactions have been identified in rice, where *OsDREB2B2*, together with NF-YA2, NF-YB3, and OsDPB3 proteins, activates the HSFA3 promoter, indicating strong evolutionary conservation of this transcriptional network among higher plants [38]. Notably, overexpression of DPB3-1 significantly improves thermotolerance in rice without compromising plant growth or yield, highlighting its potential utility in breeding programs aimed at developing heat-resilient cultivars.

Another key regulator, MBF1c, acts as an upstream transcriptional co-activator that rapidly accumulates during heat stress and binds CTAGA motifs in the promoters of downstream TFs, including DREB2A, HSFB2a, and HSFB2b, thereby facilitating rapid transcriptional reprogramming under elevated temperatures [40]. Beyond transcriptional regulation, heat stress also stimulates ROS production and transient Ca^2+^ signaling, which interact with MAPK cascades and phytohormonal pathways, particularly ABA, to coordinate HSF activation, antioxidant defense, protein quality control, and cellular homeostasis. The induced HSPs, including HSP17.7, HSP18.2, HSP21, HSP83.1, and HSP101, function as molecular chaperones that stabilize proteins, prevent irreversible protein aggregation, and facilitate protein refolding, thereby preserving cellular integrity under high-temperature conditions.

Phytochrome B (phyB) is one of the best-characterized thermosensory components in Arabidopsis, particularly in the regulation of temperature-responsive development. The phyB–ELF3–PIF4 thermosensory framework described below has been mechanistically established primarily in *Arabidopsis thaliana* and is included here as a reference model for temperature perception. These mechanisms should not be interpreted as fully validated rice pathways unless corresponding components have been functionally demonstrated in *Oryza sativa* [41]. Thermal reversion of phyB from its active Pfr state to the inactive Pr state can alter photobody dynamics and downstream signaling under specific light and photoperiod conditions. Similarly, the Evening Complex component ELF3 can respond to elevated temperature through changes in its condensation and interaction with PIF4, thereby contributing to temperature-dependent developmental responses. PIF4 subsequently integrates temperature and light signals to regulate thermomorphogenic growth [42].

Recent studies show that phyB-mediated thermosensing persists under moderate daylight and is abolished only under strong red light (≥50 μmol m^−2^ s^−1^). Furthermore, temperature-response analyses reveal a light-repressible phyB-dependent response at 12–21 °C and a phyB-independent response at 21–27 °C, indicating the involvement of additional thermosensory mechanisms at higher temperatures [42]. EARLY FLOWERING 3 (ELF3), a core component of the Evening Complex (EC) that also contains ELF4 and LUX ARRHYTHMO, functions as a transcriptional repressor of the growth-promoting factor PIF4. ELF3 thermosensing operates through a mechanistically distinct principle: temperature-induced liquid–liquid phase separation.

ELF3 contains a prion-like domain (PrLD) an intrinsically disordered region that confers the capacity for temperature-dependent phase transitions. At elevated temperatures, ELF3 forms nuclear liquid droplets through phase separation, which temporarily disrupts its transcriptional repressor function and de-represses PIF4 transcription. The Evening Complex represses PIF4 transcription specifically in the early night under short-day conditions, suggesting ELF3 senses temperature primarily at night [43].

A key thermoresponsive regulator, PHYTOCHROME INTERACTING FACTOR 4 (PIF4), induces GIGANTEA (GI) expression, while GI subsequently represses PIF4 activity through a partially TOC1-dependent mechanism, forming a negative feedback loop that fine-tunes thermomorphogenesis. This regulatory circuit is controlled by temperature-dependent inactivation of ELF3 and HY5 and involves H2A.Z-mediated chromatin remodeling at the GI locus, linking transcriptional and epigenetic regulation of plant temperature responses [44]. However, recent studies demonstrate that in long-day and continuous light conditions, ELF3-independent pathways operate to enable thermomorphogenesis. High temperatures trigger starch breakdown and sucrose production, which stabilizes PIF4 by antagonizing phyB-mediated degradation in Arabidopsis. In parallel, ELF3 inhibition releases repression of PIF4 transcription and activity. This multisensor framework explains how plants respond to high temperatures during daylight through concerted action of chloroplast-sucrose-mediated and ELF3-dependent pathways [42].

In rice, heat tolerance is more directly associated with rapid Ca^2+^ and ROS signaling, activation of heat-shock transcription factors (HSFs), induction of molecular chaperones and heat-shock proteins (HSPs), antioxidant defense, and maintenance of cellular protein homeostasis. Rice HSFs, including HSFA1-, HSFA2-, and HSFB-related factors, regulate downstream heat-responsive genes and HSPs, while additional regulators such as DREB2A, NF-Y, MBF1, NAC, and MAPK-associated components contribute to heat-responsive signaling. Importantly, rice heat responses are strongly dependent on tissue and developmental stage. Reproductive tissues are particularly vulnerable, with heat during flowering affecting spikelet fertility, reproductive development, grain filling, and yield. Therefore, mechanisms underlying reproductive-stage heat tolerance should be considered separately from temperature-induced developmental responses described in Arabidopsis. Anther and pollen protection, maintenance of fertilization capacity, protein homeostasis, ROS detoxification, and recovery after heat exposure represent important components of this response and should receive greater emphasis when discussing agronomically relevant heat tolerance in rice. Thus, the phyB–ELF3–PIF4 thermosensory framework described above is well established in Arabidopsis but should not be interpreted as a fully validated rice pathway; in rice, direct evidence is stronger for Ca^2+^/ROS signaling, HSF–HSP regulation, antioxidant defense, and associated transcriptional networks. Thus, phyB-, ELF3-, and PIF4-based thermosensing should be considered *Arabidopsis*-derived mechanistic evidence, whereas direct evidence in rice is stronger for Ca^2+^/ROS signaling, HSF–HSP regulation, antioxidant defense, protein homeostasis, and reproductive-stage heat responses [45].

Overall, early heat perception, Ca^2+^ and ROS signaling, HSF-mediated transcriptional regulation, molecular chaperone activity, and hormonal crosstalk all work together to maintain cellular homeostasis and guarantee plant survival during heat stress. This suggests that thermotolerance is controlled by a highly integrated signaling network. It will be easier to create rice cultivars that are more resilient to rising global temperatures and other co-occurring abiotic stresses if the relationships between these regulatory mechanisms are clarified.

### 4.3. Drought Stress Sensing and Signal Transduction

**Figure 3:**
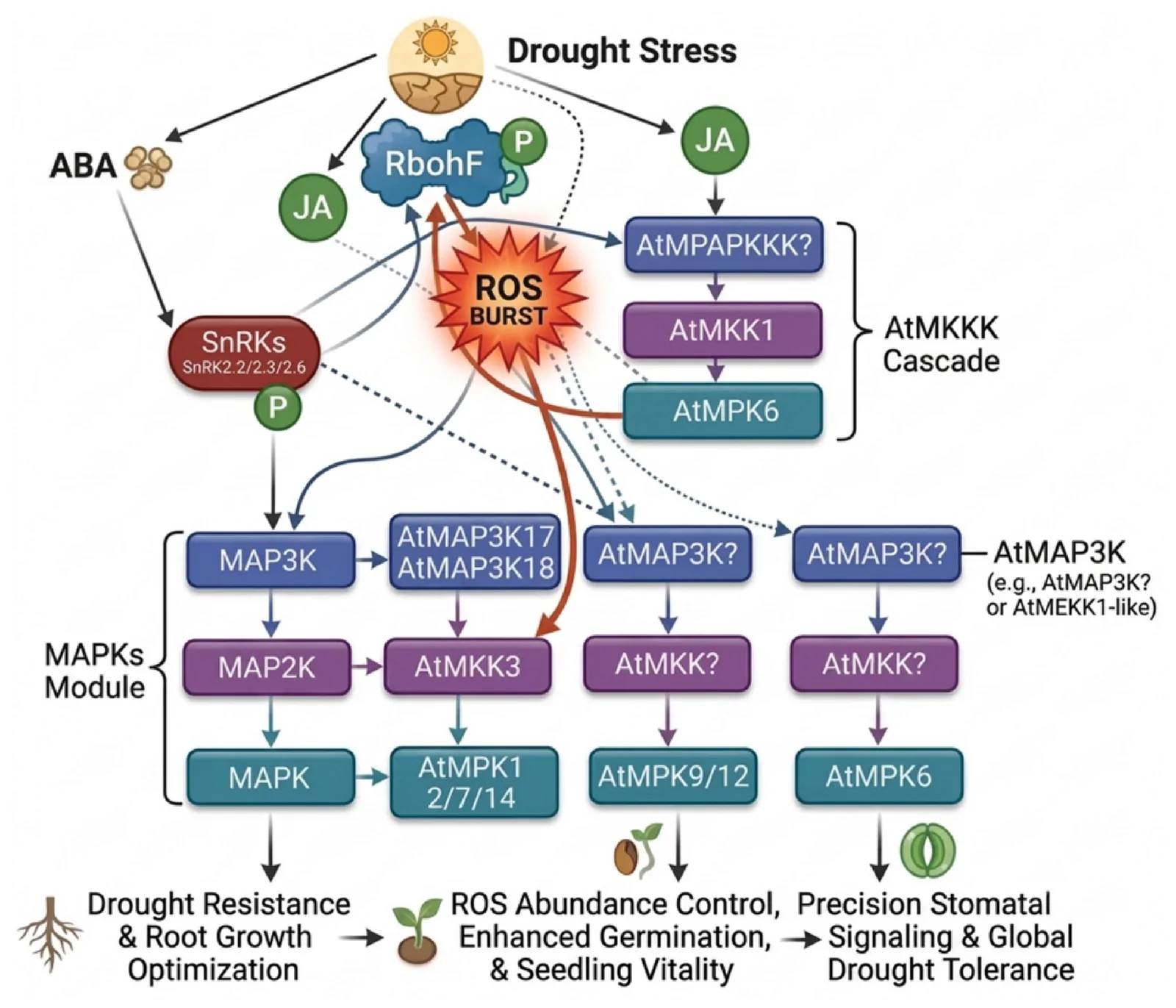
*Arabidopsis*-derived reference model of MAPK, ABA, ROS, and Ca^2+^ signaling under drought stress. The MAP3K17/18–MKK3–MPK1/2/7/14, MPK9/12, RbohF, and MKK1–MPK6 modules shown in this schematic have been functionally characterized primarily in *Arabidopsis thaliana* and are presented as comparative mechanistic frameworks rather than established rice pathways. Rice-specific drought mechanisms are discussed separately in Section 4.3 and are limited to components supported by direct evidence in *Oryza sativa*. Solid arrows indicate experimentally demonstrated interactions in the species indicated, whereas dashed arrows represent indirect or proposed relationships. Solid arrows represent established direct activation or downstream signaling pathways, dashed arrows indicate hypothesized or indirect links, and green circles labeled “P” denote phosphorylation events. Created in BioRender. NURUL, M. (2026) https://BioRender.com/urn3wnk.

Drought is one of the most serious environmental factors limiting rice growth and productivity, which mainly affects plant development, reduces photosynthesis, and disrupts cellular homeostasis. Plants have developed complex sensing and signaling networks that rapidly detect changes in cellular water status and coordinate physiological, biochemical, and molecular responses to survive water-deficient conditions (Figure 3). In contrast to salt stress, dryness is first detected by decreases in cell turgor, membrane tension, and osmotic potential. These changes trigger mechanosensitive ion channels and osmosensors, which cause the concentration of Ca^2+^ in the cytosol to rise quickly. As secondary messengers, these early calcium fingerprints initiate several downstream signaling pathways that are responsible for drought adaptation.

One of the principal regulatory mechanisms governing drought responses is the abscisic acid (ABA)-dependent signaling pathway. Water deficit stimulates ABA biosynthesis, resulting in its accumulation in guard cells and other tissues. ABA is perceived by PYRABACTIN RESISTANCE/PYR-LIKE/REGULATORY COMPONENTS OF ABA RECEPTORS (PYR/PYL/RCARs), which inhibit protein phosphatase 2C (PP2C) proteins such as ABI1 and ABI2. This inhibition releases SNF1-related protein kinase 2 (SnRK2) proteins from PP2C-mediated suppression, allowing them to phosphorylate downstream transcription factors, ion channels, and stress-responsive proteins that regulate stomatal closure, osmotic adjustment, and drought-responsive gene expression. MAPK cascades constitute another major signaling pathway that integrates drought perception with downstream adaptive responses. MAPK signaling consists of a sequential phosphorylation cascade involving MAPK kinase kinase (MAPKKK), MAPK kinase (MAPKK), and MAPK, enabling efficient amplification and transmission of extracellular stress signals [46,47,48]. Activated MAPKs phosphorylate numerous downstream targets, including transcription factors, cytoskeletal proteins, phospholipases, and protein kinases, thereby regulating diverse cellular processes such as cell division, growth, hormone signaling, programmed cell death, and stress adaptation [49]. The following MAPK module has been functionally characterized primarily in Arabidopsis and is included here as a mechanistic reference framework. Its conservation and functional equivalence in rice require confirmation unless corresponding Os homologs have been directly validated.

The MAP3K17/18–MKK3–MPK signaling module described below has been functionally characterized primarily in *Arabidopsis thaliana*. It is therefore presented as a mechanistic reference framework and should not be regarded as an established rice drought-signaling pathway unless functional conservation of the corresponding rice components has been demonstrated experimentally [50,51]. Activated MAP3K17/18 subsequently phosphorylates MKK3, which in turn activates MPK1, MPK2, MPK7, and MPK14, forming the ABA-responsive MAP3K17/18–MKK3–MPK signaling module that regulates drought-responsive gene expression and stress adaptation [52]. Functional studies have demonstrated that MAP3K18 also participates in stomatal development and ABA-induced stomatal closure. Arabidopsis map3k18 mutants exhibit altered stomatal density, increased stomatal size, and impaired ABA-mediated stomatal regulation, resulting in enhanced susceptibility to drought stress [50]. Furthermore, ABA-induced activation of MAP3K17 and MAP3K18 has been shown to strengthen downstream stress signaling, emphasizing their central role in coordinating drought tolerance [53,54].

During drought stress, ROS works closely with the ABA and MAPK pathways. Stomatal closure, antioxidant defense, and stress-responsive gene expression are all regulated by controlled ROS generation, which is mainly mediated by RESPIRATORY BURST OXIDASE HOMOLOGS (RBOHs) such as AtRbohF [53]. Through reciprocal positive-feedback pathways, wherein Ca^2+^ activates RBOH proteins and ROS further promotes Ca^2+^ influx, drought-induced signaling is intimately interwoven with Ca^2+^ signaling. Numerous transcription factor families, such as DREB, AREB/ABF, NAC, WRKY, MYB, and bZIP, are subsequently activated by these interrelated pathways, coordinating the expression of genes related to osmoprotectant biosynthesis, antioxidant defense, cellular protection, and water conservation. Also, AtRbohF has been determined to communicate with Ca^2+^ signaling and improve Arabidopsis tolerance to oxidative stress [55]. In rice, direct evidence more strongly supports drought-induced ABA signaling, stomatal regulation, ROS homeostasis, osmotic adjustment, antioxidant defense, and activation of stress-responsive transcription factors. Therefore, *Arabidopsis*-derived MAPK and RBOH modules are discussed here only as comparative models, whereas rice-specific conclusions are restricted to mechanisms supported by direct studies in *Oryza sativa*. Accordingly, AtRbohF-based ROS–Ca^2+^ crosstalk should be considered evidence from Arabidopsis rather than direct demonstration of the same regulatory module in rice.

Collectively, drought tolerance is governed by an integrated signaling network in which osmotic stress perception, Ca^2+^ signaling, ABA signaling, MAPK cascades, ROS signaling, and transcriptional regulation function cooperatively to maintain cellular homeostasis and improve plant survival under water-deficit conditions. A comprehensive understanding of the interactions among these signaling pathways will facilitate the identification of key regulatory genes and support the development of drought-resilient rice cultivars through molecular breeding and genome-editing approaches.

## 5. Physiological and Regulatory Components Underlying Multi-Stress Responses

### 5.1. Morpho-Physiological Responses to Different Abiotic Stresses

**Figure 4:**
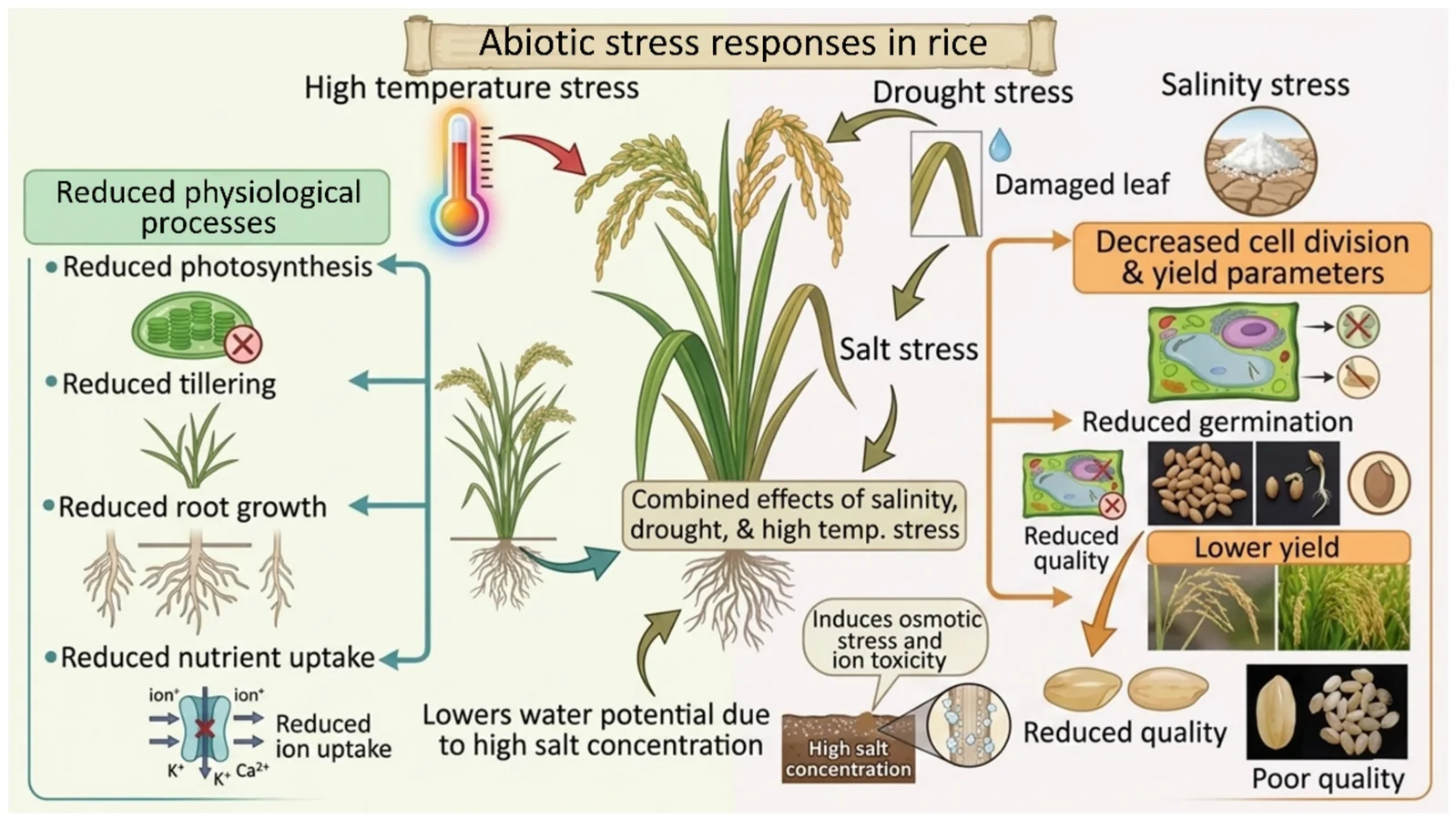
Physiological consequences of heat, drought, salinity, and their combinations in rice. The schematic links each stress to experimentally measurable physiological disruptions, including altered water relations, ion homeostasis, reactive oxygen species (ROS) accumulation, photosynthetic inhibition, reproductive damage, and yield reduction. Shared responses are distinguished from stress-specific effects to highlight convergent and combination-specific mechanisms. Created in BioRender. NURUL, M. (2026) https://BioRender.com/1tygg96.

Global rising temperatures contribute to the problem of high temperatures, which has become a serious challenge to securing food security worldwide. Rice crops possess varied morphological and physiological responses to heat stress. Thus, it is highly necessary to create rice cultivars that are resistant to high temperatures to enhance the quality and productivity of rice crops, which are otherwise being degraded by high temperatures [33] and other abiotic stresses (Figure 4). As soil temperature increases, it has been observed that the growth of roots and their physiological activity and shoot growth decrease [56]. High temperature (42–45 °C) during the early development of plants causes severe water loss, which results in wilting and yellowing of the leaves. It reduces seedling and root growth, and in extreme cases, even burns the seedlings [57]. It is anticipated that every 1 °C rise in average global temperature will cause global reductions in cereal grain yield on major cereal crops [58]. Ideal growth of rice occurs between 27 and 32 °C; meanwhile, the growth and development of rice plants are negatively impacted by temperatures above 32 °C [59].

Heat stress in plants is detrimental to the normal operations of proteins and nucleic acids and therefore influences plant thermotolerance. Oxidative stress is due to high temperatures that cause the dissociation of the oxygen-evolving complex (OEC) in photosystem II (PSII), that decrease the transfer of electrons between the OEC and the acceptor side of the PSII. In addition, the overall experience is that drought conditions in rice plants may induce the inactivation of enzyme catalysis, the formation of growth promoters, and modifications in source-sink relationships [60]. Water shortage also negatively influences many physiological characteristics surrounding rice, such as net photosynthetic rate, the rate of transpiration, stomatal conductance, the efficiency of water utilization, the internal atmospheric CO_2_ level, activity rate of PSII, the degree of moisture, and the integrity of the membrane [61]. Plant root traits also play a decisive role in crop yield in drought. Unlike its vegetative stages, rice is prone to drought during its reproductive stages [62]. Additionally, drought stress induces the alteration of membrane transport, poor formation of Adenosine Triphosphate (ATP), and poor seed development [63].

Salt stress in rice imposes both osmotic and ionic constraints. The initial osmotic component reduces water availability and cellular turgor, thereby restricting root and shoot growth, leaf expansion, and photosynthetic activity. With prolonged or severe salinity, excessive Na^+^ uptake and transport can disturb cellular ion homeostasis, particularly the K^+^/Na^+^ ratio, and can interfere with nutrient acquisition, enzyme activities, membrane function, and metabolic processes. Rice therefore employs coordinated mechanisms to restrict Na^+^ entry and transport, extrude Na^+^ from the cytosol, and sequester excess Na^+^ into vacuoles [64]. Ionic stress, conversely, causes an enormous infusion of sodium into the rice crop, resulting in a high concentration of salt in the mature leaves, which reaches a dangerous level. That leads to a high concentration of Na^+^ in the nuclear membrane that interferes with cell metabolism and eventually eradicates cells [65]. Research by Cha-umi et al. Showed, there was a substantial reduction in flavonoid and chlorophyll in rice leaves as a result of salt stress [66]. High drought and salinity stresses can activate the presence of ROS that, consequently, influence several morpho-physiological and yield-related traits by causing oxidative stress [67].

### 5.2. Biochemical Responses to Different Abiotic Stresses

Abiotic stresses induce deep alterations in cellular activities, resulting in a significant impact on plant physiology. Several ineffective strategies are direct consequences of abiotic-stress damage. Abnormal changes in the structure of protein and protein structure and membrane mobility also occur due to stressors, including heat, drought, and salt stress. It has been demonstrated that rice possesses an abundance of genes linked to high temperature, including stress-related TFs, heat stress transcription factors (HSTFs), and HSPs, which have been successfully characterized. Scientists have identified 16 whole CNGC genomes in rice. Among them, 13 are present in the plasma membrane and are believed to be subject to temperature changes [68]. Concerning HS, the correlation amongst Ca^2+^ relay, as well as the ROS regulatory network in particular, is that the excessive generation of H_2_O_2_ is extensively explained [69]. H_2_O_2_ is also able to cause a marked elevation in cytosolic levels of Ca^2+,^ and this is through the subsequent activation of the Ca^2+^ signaling pathway. This pathway has a direct connection to the origin of the ROS by respiratory burst oxidase homolog D (RBOHD) [70].

HSTFs are important in the stimulation of genes that code for HSF-stimulated TFs, ROS-scavenging enzymatic agents, metabolic enzymes, and HSPs [71]. Some of the protein molecules located in the membranes can perceive changes in the structural properties of the cellular membranes. The absence of functional mutations in *OsCNGC9*, *OsCNGC14*, and *OsCNGC16* results in a reduced ability of rice to respond to elevated temperature. This eventually reduces the capability of rice to resist different abiotic stresses. Heat stress and cold stress may cause changes in protein integrity, and heat stress can be characterized in any part of the cells. When water shortage occurs, stomata close, and eventually this causes high temperatures.

Both drought and heat stress suppressed the expression of genes that code POD and photosynthetic proteins and stimulated CAT, other proteins, and TF [72]. Rice under drought stress activates ABA-dependent and -independent signaling. ABA controls the opening and closing of stomata to slow down transpiration when there is drought stress [73]. Drought stress may cause ROS to accumulate in rice. Lipids in the membrane, mostly malondialdehyde (MDA), can become degraded due to excessive ROS formation and accumulation. Water deprivation increases the action of SOD and CAT in rice and, consequently, inhibits MDA formation [74]. DST, a zinc transcription factor, is a repressor of rice to withstand drought. It increases the activation of genes that are linked to ROS and ultimately determines drought tolerance in rice [75]. Important roles of bZIP, ERF, WRKY, and NAC TFs are related to the transfer and reaction of drought stress-related signals.

The SOS pathway is crucial to control the balance of ions in a cell as a result of salt stress [76]. TFs may have either beneficial or detrimental effects on salt tolerance in rice plants under different conditions. These alterations involve an accumulation of osmo-protectants and antioxidants and an increase in the efficiency of Na^+^ and K^+^ channels [77]. Wang et al. have singled out three particular genes, i.e., *OsHKT1;1*, *OsHAK10*, and *OsHAK16*, which proved important in promoting the migration of sodium ions (Na^+^) in the roots to the shoot. The process of enzyme reaction is controlled through Na^+^/H^+^ antiporters, which are able to transfer Na^+^ ions from higher concentration to lower concentration through the use of proton pumps [78]. Besides drought, salinity enhances the excessive accumulation of ROS, which is central to phytohormones and the expression of various genes in the signaling pathway. A number of molecular studies have examined how the rice plant can react to higher salt stress conditions and can become valuable in understanding how to combine these particular genes into future breeding programs.

### 5.3. Hormonal Responses to Abiotic Stresses

Phytohormone responses in rice are strongly dependent on the nature, intensity, duration, tissue, and developmental stage of stress. Rather than acting as independent pathways, ABA, auxin, ethylene, jasmonates, cytokinins, gibberellins, brassinosteroids, salicylic acid, and strigolactones interact to balance growth, water conservation, ion homeostasis, and reproductive development. However, the available rice evidence indicates that these interactions are not equivalent under different stress combinations. In particular, combined heat and drought impose a strong reproductive and carbon-allocation constraint, whereas drought combined with salinity places greater emphasis on water conservation, ionic/osmotic adjustment, and root–shoot coordination.

**Figure 5:**
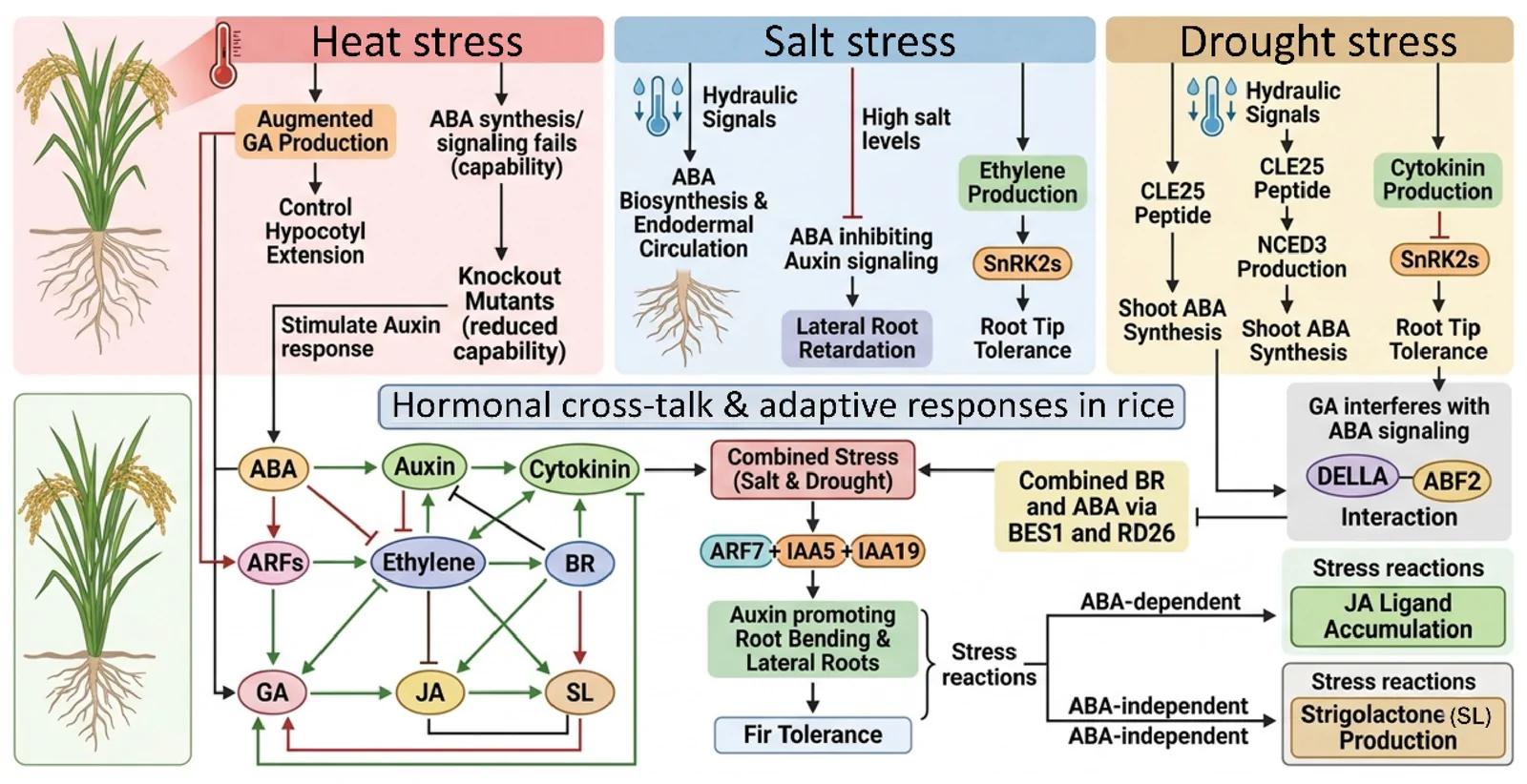
Integrated phytohormone signaling and adaptive crosstalk under abiotic stress in rice. The schematic summarizes interactions among abscisic acid (ABA), auxin, ethylene (ET), jasmonic acid (JA), cytokinins (CKs), gibberellins (GAs), brassinosteroids (BRs), salicylic acid (SA), and strigolactones (SLs) during heat, salinity, and drought responses. Arrows indicate interactions directly demonstrated in rice or rice homologs from other plant species, and T-bars indicate inhibition or repression. Each interaction is supported by the primary references cited in Section 5.3. Created in BioRender. NURUL, M. (2026) https://BioRender.com/lbecpkd.

Phytohormonal crosstalk is an important component of rice thermotolerance. Integrated phytohormone signaling and adaptive crosstalk under abiotic stress in rice have been demonstrated in Figure 5. ABA promotes heat tolerance through antioxidant defense and heat-responsive gene regulation, while auxin, brassinosteroids (BRs), cytokinins (CKs), salicylic acid (SA), jasmonates (JAs), and ethylene (ET) regulate photosynthesis, ROS homeostasis, growth, and reproductive development under high temperature. In rice, OsNCED1-mediated ABA biosynthesis and BR-associated regulators contribute to thermotolerance, whereas heat-induced changes in ABA, IAA, JA, CKs, and other hormones vary with genotype and developmental stage. These findings indicate that rice heat tolerance depends on coordinated hormone crosstalk rather than the action of a single hormonal pathway [79]. ABA represents an important convergence point during drought and salinity because water deficit and osmotic stress stimulate ABA accumulation and downstream signaling that restricts water loss and adjust metabolism. In rice, genes involved in ABA biosynthesis and signaling, including OsNCED2 and ABA-responsive regulatory components, contribute to drought adaptation [80,81]. However, increased ABA signaling can also suppress growth processes, illustrating a trade-off between stress survival and biomass production. This trade-off is particularly relevant when drought occurs during vegetative growth, whereas reproductive-stage drought requires maintenance of carbon assimilation and reproductive development in addition to water conservation. Under combined heat and drought, ABA-dependent stomatal regulation must also be considered together with heat-induced changes in photosynthesis, carbon metabolism, and reproductive fertility rather than being interpreted solely as a drought-response pathway. Rice studies of combined drought and heat demonstrate substantial hormonal changes in flag leaves and spikelets and indicate that the hormonal response differs among genotypes [82].

Auxin signaling undergoes extensive reprogramming during stress conditions to optimize root system architecture and water acquisition. Auxin promotes lateral root formation and root bending through the coordinated action of AUXIN RESPONSE FACTOR 7 (ARF7) and its interacting repressors IAA5 and IAA19, thereby enhancing plant adaptation to drought and salinity [83]. However, elevated ABA levels suppress auxin signaling in the root endodermis, restricting lateral root development under severe stress and prioritizing survival over growth. Heat stress also influences auxin homeostasis and signaling, contributing to developmental adjustments required for thermal adaptation [84].

Other phytohormones function cooperatively or antagonistically with ABA to fine-tune stress responses. Brassinosteroids (BRs) enhance tolerance to multiple abiotic stresses through interactions between the transcription factor BES1 and the stress-responsive regulator RD26, thereby coordinating growth with defense responses [85]. Gibberellins (GAs) generally promote plant growth, whereas stress-induced ABA signaling suppresses GA responses through the stabilization of DELLA proteins, which interact with transcription factors such as ABF2 to redirect cellular resources toward stress tolerance. This ABA–GA antagonism represents a key regulatory mechanism balancing growth and survival during drought and salinity stress.

Stress adaptation also involves coordinated interactions with jasmonic acid (JA), ethylene (ET), cytokinins (CKs), salicylic acid (SA), and strigolactones (SLs). JA accumulates rapidly under abiotic stress and cooperates with ABA to activate antioxidant defenses, osmoprotectant biosynthesis, and stress-responsive gene expression. Ethylene contributes to stress acclimation by regulating root development, senescence, and ROS homeostasis, whereas cytokinin signaling is generally downregulated during drought to reduce growth and conserve resources. Strigolactones further modulate root system architecture and interact with ABA-dependent and -independent pathways to improve drought resilience. Collectively, these hormones establish a highly coordinated regulatory network that integrates developmental signals with environmental cues.

Salicylic acid (SA) has become a significant regulator of abiotic stress tolerance through the regulation of redox homeostasis, antioxidant defense, and stress-responsive signaling networks. SA limits oxidative damage and maintains membrane integrity by increasing the activity of antioxidant enzymes, including SOD, CAT, and POD, during drought, salinity, and heat stress. Recent research also shows that SA controls stress adaptation by coordinating stomatal regulation, photosynthetic efficiency, osmotic adjustment, and transcriptional reprogramming under unfavorable environmental conditions through extensive crosstalk with ABA, JA, ET, and MAPK signaling pathways. Furthermore, SA is a crucial part of integrated defense systems because it activates both NPR1-dependent and NPR1-independent signaling pathways, which improves cellular defense against various abiotic stressors [86,87,88].

Cytokinins (CKs) are key regulators of cell division, shoot development, nutrient allocation, and source–sink relationships. Although endogenous cytokinin levels generally decline during drought and salinity, stress-induced modulation of CK biosynthesis and signaling helps optimize the balance between growth and survival. CKs delay leaf senescence, maintain chloroplast structure and photosynthetic capacity, regulate root–shoot communication, and improve nutrient remobilization under stress conditions. Increasing evidence also indicates that CK signaling interacts closely with ABA, SnRK2 kinases, ROS signaling, and auxin pathways to coordinate stomatal behavior, root architecture, and antioxidant defense during abiotic stress adaptation. Manipulating cytokinin metabolism or signaling components, such as ISOPENTENYL TRANSFERASE (IPT) and CYTOKININ OXIDASE/DEHYDROGENASE (CKX) genes, has shown considerable promise for enhancing drought and salinity tolerance while minimizing growth penalties, highlighting CK signaling as an attractive target for climate-resilient crop improvement [89,90].

Extensive crosstalk between phytohormones and other signaling pathways, including Ca^2+^ signaling, ROS, MAPK cascades, and stress-responsive TFs, ensures precise regulation of gene expression and cellular homeostasis under fluctuating environmental conditions. Deciphering these interconnected hormonal networks will provide valuable insights into the molecular basis of stress tolerance and facilitate the development of climate-resilient rice cultivars through modern breeding and genome-editing strategies.

### 5.4. Impact of Abiotic Stresses on Rice Yield

Rice productivity is increasingly affected by climate-related abiotic stresses, particularly drought, salinity, and high temperature, with the magnitude of their effects varying among rice-growing environments and developmental stages [91,92,93]. Drought can substantially reduce photosynthesis, biomass accumulation, and water-use efficiency during vegetative growth, whereas water deficit during reproductive development is particularly detrimental because it can delay flowering, reduce pollen viability, increase spikelet sterility, impair grain filling, and ultimately decrease grain yield [94]. These effects are associated with altered carbohydrate metabolism, excessive ROS accumulation, impaired water relations, and ABA-mediated stomatal regulation.

Salinity is the second major abiotic stress limiting rice productivity, particularly in coastal and irrigated regions. Salt stress induces osmotic stress, ion toxicity, nutrient imbalance, and oxidative damage, resulting in reduced photosynthesis, impaired panicle development, decreased spikelet fertility, and deterioration of grain quality [95,96]. Grain quality is further reduced by increased chalkiness and reduced milling quality under saline conditions [97]. Temperature stress profoundly affects rice growth, especially during the reproductive stage. Cold stress impairs pollen development and fertilization, whereas heat stress accelerates leaf senescence, disrupts starch biosynthesis, increases spikelet sterility, and reduces grain yield and grain quality [98,99,100]. Exposure to temperatures above 33 °C during flowering can reduce grain yield by approximately 24–27% [100].

In natural field, rice is frequently exposed to combined stresses, which generally impose greater yield penalties than individual stress factors by simultaneously disrupting photosynthesis, hormonal balance, reproductive development, and cellular homeostasis. Therefore, developing climate-resilient rice cultivars with enhanced tolerance to multiple abiotic stresses through integrated physiological, molecular, and breeding approaches is essential for sustaining future rice production and ensuring global food security.

## 6. Rice Responses to Combined Abiotic Stresses: Interaction-Specific Mechanisms

The understanding of rice responses to combined abiotic stresses is critical for developing climate-resilient varieties, as these responses are distinct from those triggered by individual stresses [101]. Demonstrated that combined drought–heat and drought–salinity stresses induce unique physiological and molecular adaptations in rice. Tolerant genotypes such as N22 (for drought + heat) and Pokkali (for drought + salinity) exhibited lower oxidative damage and higher expression of antioxidant and stress-responsive genes, including CAT, APX, SOD, DEHYDRIN, and GATA28. In contrast, sensitive genotypes showed greater ROS accumulation, increased membrane damage, and reduced photosynthetic efficiency, underscoring the necessity of breeding for tolerance to specific stress combinations that activate distinct adaptive mechanisms. The shared involvement of Ca^2+^, ROS, ABA, MAPK cascades, or common transcription factors across individual stresses does not by itself demonstrate functional crosstalk under combined stress. Such interactions should be regarded as putative until validated in experiments that directly compare individual and combined treatments. Combined-stress effects can be classified as additive, synergistic, or antagonistic according to whether the combined response equals, exceeds, or is weaker than the expected effect of the component stresses [102].

Several genes have been identified as positive regulators of stress tolerance across different species. For instance, the rice transcription factor SNAC1, when transgenically expressed in wheat, significantly enhanced tolerance to both drought and salinity. Transgenic wheat plants exhibited improved leaf water content, chlorophyll retention, and increased root biomass compared to wild-type controls. This enhanced tolerance was associated with increased ABA sensitivity and the regulation of multiple ABA- and stress-responsive genes, highlighting the potential of SNAC1 for genetic engineering of abiotic stress tolerance in cereals [103]. Additionally, the rice small heat shock protein gene OsHSP16.9 is rapidly and strongly induced by high salinity, osmotic stress (simulating drought), and heat. Its overexpression has been shown to confer enhanced tolerance to both salt and drought stresses in transgenic rice [103].

Conversely, certain genes function as negative regulators of stress tolerance, representing potential targets for knockout or suppression strategies. The rice E3-ubiquitin ligase OsDHSRP1, characterized by a C3HC4 zinc finger domain, is highly induced by salinity, heat, and drought. It promotes ROS production, thereby functioning as a negative modulator of stress responses and representing a potential point of vulnerability in the stress defense network [104]. Similarly, the *OsDSR2* (*DUF966*-stress repressive gene 2) gene has been conclusively shown to negatively regulate salt and drought tolerance in rice. RNAi-mediated knockdown of OsDSR significantly enhanced stress resistance, accompanied by reduced oxidative damage, evidenced by decreases in cell membrane permeability (~3-fold), MDA content (~3.5-fold), O_2_•− (~20%), and H_2_O_2_ (~10.5%) [105]. In maize, the heat shock transcription factor ZmHSF08 also acts as a negative regulator, as its overexpression increased stress sensitivity through elevated ROS and MDA accumulation and reduced plant survival, which was linked to the downregulation of ABA- and stress-related *ZmDREB2A*, *ZmRAB18*, *ZmERD1*, *ZmNHX3*, and *ZmLEA2* genes [106]. Collectively, these findings illustrate the complex regulatory networks governing abiotic stress tolerance in plants, involving both positive and negative regulators. Combined-stress responses cannot be interpreted solely from the identities of the component stresses, because the biological outcome also depends on the order, duration, and developmental timing of exposure. Simultaneous stresses activate overlapping signaling networks at the same time and may generate competition or reinforcement among protective responses, whereas sequential stresses allow the first stress to alter the physiological and transcriptional state in which the second stress is perceived. Similarly, acute exposure primarily captures rapid signaling and cellular protection, whereas chronic exposure additionally involves acclimation, growth trade-offs, metabolic adjustment, and cumulative damage. Developmental stage is equally important: vegetative-stage responses largely determine maintenance of growth, photosynthesis, and water relations, whereas reproductive-stage combinations directly threaten flowering, pollen viability, fertilization, grain filling, and yield formation. Therefore, comparisons among combined-stress studies should account for exposure mode, duration, developmental stage, genotype, and stress intensity before classifying an interaction as additive, synergistic, or antagonistic. Table 1 summarizes comparative synthesis of rice responses to combined abiotic stresses.

### 6.1. Combined Heat and Drought Stress

The combined heat and drought stress strongly restrict photosynthesis and carbon allocation through interacting effects on stomatal closure, thermal damage, respiration, ROS production, and protein stability. The combination is especially damaging during reproductive development, reducing pollen viability, spikelet fertility, grain filling, and yield. ABA, Ca^2+^–ROS signaling, antioxidant defenses, heat-shock factors, and molecular chaperones jointly regulate the response, although ABA-induced stomatal closure may reduce transpirational cooling under heat. Rice responses are also genotype dependent, with tolerant genotypes showing lower oxidative damage and stronger antioxidant and stress-responsive gene activity under combined drought and heat [107].

### 6.2. Combined Drought and Salinity Stress

The combined drought and salinity stress cause both osmotic stress and ion toxicity, requiring coordinated regulation of water conservation, Na^+^ exclusion, K^+^/Na^+^ homeostasis, antioxidant defense, and growth. The severity of damage depends on stress intensity, duration, and exposure sequence. Rice tolerance is strongly genotype dependent; tolerant genotypes such as Pokkali show stronger antioxidant and stress-responsive capacity, indicating that combined-stress tolerance should be evaluated directly rather than inferred from single-stress performance [101].

### 6.3. Combined Heat and Salinity Stress

The combined heat and salinity stress in rice remains relatively understudied. Heat-induced membrane and protein damage can interact with salinity-driven osmotic and ionic stress, jointly impairing photosynthesis, redox balance, and reproductive development. Shared Ca^2+^, ROS, ABA, antioxidant, and heat-shock pathways may provide partial protection, but the overall response depends strongly on genotype, stress severity, duration, and developmental stage [108]. Combined-stress responses should be evaluated using factorial experiments that compare each individual stress with the corresponding combined treatment under matched conditions. This allows additive, synergistic, and antagonistic interactions to be distinguished and helps determine whether shared signaling components such as Ca^2+^, ROS, ABA, MAPKs, and transcription factors functionally interact under combined stress [102].

## 7. Strategies to Combat Abiotic Stresses

### 7.1. CRISPR/Cas9 Gene-Editing Technology

By interfering with physiological, metabolic, and molecular processes necessary for plant growth and development, abiotic stressors significantly reduce rice output [95,96]. As a result, creating rice cultivars that are resistant to climate change has emerged as a key breeding goal. Through targeted mutation of stress-responsive genes, the CRISPR/Cas9 genome-editing system has become a potent and accurate tool for enhancing abiotic stress tolerance among contemporary biotechnological techniques [71]. In contrast to traditional breeding, CRISPR/Cas9 allows for the quick and effective editing of particular genomic loci, hastening the creation of better rice varieties. CRISPR/Cas9 has been successfully applied for both single-gene and multiplex genome editing in several crops, including rice, wheat, maize, and cotton, enabling the simultaneous manipulation of multiple stress-responsive genes involved in drought, salinity, and heat tolerance [109,110]. CRISPR/Cas systems have been successfully established for targeted genome modification in rice; however, proof of editing efficiency should be distinguished from functional improvement of abiotic-stress tolerance. For example, OsPDS has been widely used as a visible editing control because disruption of this gene produces an easily detectable albino or photobleaching phenotype; therefore, OsPDS editing demonstrates genome-editing functionality rather than enhanced abiotic-stress tolerance. Early studies establishing CRISPR/Cas-mediated mutagenesis in rice should likewise be regarded as proof-of-concept demonstrations unless stress tolerance was directly evaluated using appropriately edited lines. Table 2 summarizes representative CRISPR/Cas9 targets for which abiotic-stress responses have been functionally evaluated. Accordingly, we consider only rice targets supported by primary functional studies in which the edited genotype was exposed to defined abiotic stress and evaluated for stress-related phenotypes as validated stress-tolerance targets [111].

The CRISPR toolkit has been further extended beyond traditional gene knockdown techniques by recent developments. Stress-responsive genes can be precisely regulated or modified without causing significant genomic changes with technologies like CRISPR activation (CRISPRa), CRISPR interference (CRISPRi), base editing, and prime editing. These cutting-edge genome-editing tools provide exciting prospects for creating intricate features regulated by several genes and regulatory networks. CRISPR-based precision breeding is anticipated to be crucial in creating rice cultivars with long-lasting resistance to heat, salinity, drought, and their combined stressors when paired with genomic selection and marker-assisted breeding.

**Table 1:** Comparative synthesis of rice responses to combined abiotic stresses based on studies cited in the present review.

Genotype(s)	Stress Combination and Exposure Stage	Stress Intensity	Main Physiological and Biochemical Outcomes	Yield or Grain-Quality Outcomes	Omics Layer	Key Genes/Pathways or Regulatory Modules	Evidence and Key Knowledge Gap	Reference
N22 and Gharib (heat-response contrasts); Moroberekan and Pusa 1121 (drought-response contrasts); Pokkali and IR64 (salinity-response contrasts)	Drought + heat stress at the seedling stage	Seedlings were grown at 35 °C and withheld irrigation until 30–40% FC	Sensitive genotypes showed greater ROS accumulation (20–40%), membrane damage (8–65%), and reductions in photosynthetic efficiency (10–23%) than tolerant checks	Significant reduction in FW, DW, RL, and SL under drought and heat stress	Physiological, biochemical, and molecular analyses	Oxidative stress, membrane stability, photosynthetic performance, and stress-responsive molecular responses	Direct rice evidence. Genotype rankings under single stresses should not automatically be assumed to predict tolerance to combined stress	[101]
N22, Gharib, Moroberekan, Pusa 1121, Pokkali, and IR64	Drought + salinity stress at the seedling stage	Seedlings were treated with 200 mM NaCl and withheld irrigation until 30–40% FC	Sensitive genotypes showed increased oxidative and membrane damage and impaired photosynthetic performance	Significant reduction in FW, DW, RL, and SL under drought and salinity stress	Physiological, biochemical, and molecular analyses	ROS accumulation, membrane damage, photosynthetic efficiency, and stress-responsive molecular mechanisms	Rice evidence. Relative contributions of osmotic stress, Na^+^ toxicity, and their interaction require combination-specific analysis	[101]
N22, Dular, and Anjali	Drought + heat imposed at flowering or early grain filling under naturally high summer temperatures	Soil water potential reached −61 kPa at flowering and −57 kPa at grain filling; ambient maximum air temperature averaged 33.9–34.5 °C	Reduced panicle conductance and increased panicle tissue temperature; cultivar- and stage-dependent physiological responses	Yield reduction ranged 20–80%. For yield reduction, Dular had 73.2% under flowering stress, and N22 had 77.6% at grain filling	Agronomic and physiological measurements	Transpirational cooling, panicle temperature regulation, spikelet number, and seed set	Strong field-based rice evidence. Responses differed by genotype and developmental stage, highlighting the need to separate reproductive-stage sensitivity from vegetative stress responses	[107]
Three rice cultivars differing in drought and heat tolerance	Drought + heat stress at flowering and early grain filling	Drought and heat at flowering or early grain filling, resulting in simultaneous heat stress	Organ-specific metabolic responses in flag leaves, flowering spikelets, and developing seeds; metabolic profiles differed among cultivars	Yield and quality were reduced under combined stress; potential metabolites associated with yield and grain quality	GC–MS-based metabolomics, with correlation analysis	Stress-regulated and constitutively different metabolites associated with yield stability and grain quality	Field-based rice evidence and metabolomic evidence for genotype- and organ-specific responses under combined drought and heat	[112]
Yukinkomai (susceptible wild type) and YNU31-2-4 (salt-tolerant cultivar)	Salinity + heat applied for 10 days, followed by stress-release/recovery assessments	75 mM NaCl; 30 °C compared with 26 °C under normal-temperature treatments	Altered growth, water status, Na^+^/K^+^ balance, photosynthetic pigments, proline, lipid peroxidation, antioxidant activities, and gene expression	No grain-yield outcome; plants were assessed at the seedling stage	Targeted transcriptional analysis plus physiological and biochemical measurements	OsHSP18/OsHSP20, OsP5CS, OsMSD1, OsSOS1, OsHKT1;5, OsNHX, aquaporins, and antioxidant systems	Rice evidence, including recovery. Demonstrates that one stress can mitigate or exacerbate particular effects of another	[108]

**Table 2:** CRISPR/Cas9-targeted genes and functional validation of abiotic stress responses in rice.

Target Locus	Mutation	Genetic Background	Stress Treatment	Main Measured Phenotype	Growth or Yield Penalty	Validation Level	Reference
OsPDS	CRISPR/Cas9-mediated loss-of-function mutation	*Oryza sativa*	No abiotic-stress treatment used for tolerance validation	Visible albino phenotype used to demonstrate genome-editing activity	Not applicable to abiotic-stress tolerance	Proof-of-concept editing control; not a stress-tolerance target	[113]
OsSAPK2	CRISPR/Cas9 knockout; sapk2-1 contained a 1 bp deletion and sapk2-7 a 7 bp deletion in the third coding exon, causing frameshifts and premature termination	Japonica rice	Drought by withholding water; additional NaCl and PEG assays	Knockout lines showed greater drought sensitivity, reduced ABA responsiveness, impaired stomatal regulation and osmotic adjustment, and weaker ROS-scavenging capacity than WT	Stress tolerance was reduced, not improved; no field-level yield advantage demonstrated	Functional laboratory validation of a positive stress-response regulator; not an improvement edit	[114]
OsRR22	CRISPR/Cas9 targeted mutagenesis of the first exon; multiple indel alleles; two T-DNA-free homozygous lines selected	Elite japonica cultivar WPB106	0.75% NaCl nutrient solution applied to 2-week-old seedlings for 14 d	Mutant lines retained substantially greater shoot fresh and dry weight and plant height than WT under salinity	No significant differences from WT for plant height, flowering time, grains panicle^−1^, spikelet fertility, or yield plant^−1^ under normal field conditions	Greenhouse stress validation with field agronomic assessment	[115]
OsDST	CRISPR/Cas9 editing with two gRNAs; homozygous dstΔ184–305 mutant containing a 366 bp in-frame deletion corresponding to amino acids 184–305	Indica mega-rice cultivar MTU1010	Dehydration/osmotic stress and high salinity at seedling stage; high-salt assays included 200 mM NaCl	Mutant showed broader leaves, reduced stomatal density, increased leaf water retention, moderate osmotic-stress tolerance and strong salt tolerance	No drawback reported in the controlled validation; authors proposed the allele for improvement of drought/salt tolerance and grain yield	Laboratory/seedling stress validation; limited field/yield validation	[116]

### 7.2. Molecular Breeding

Advances in high-throughput sequencing (HTS) and molecular marker technologies have significantly accelerated the development of rice cultivars with enhanced tolerance to abiotic stresses. Marker-assisted backcrossing (MABC) is a popular molecular breeding technique that minimizes linkage drag while transferring advantageous QTLs into genetic backgrounds with high yields [117]. However, MABC alone is frequently inadequate for enhancing quantitative features since abiotic stress tolerance is usually regulated by several genes with tiny individual effects and intricate epistatic interactions [118]. Marker-assisted recurrent selection (MARS) and genomic selection (GS) have been developed to increase genetic gain for complex traits including drought, salinity, and heat tolerance by simultaneously accumulating numerous beneficial alleles [119].

The identification of superior alleles influencing stress tolerance has been further enhanced by recent developments in genomic prediction, haplotype-based breeding, and genome-wide association studies (GWAS). These methods speed up breeding efficiency and allow for more precise selection of climate-resilient rice genotypes when combined with high-throughput phenotyping and next-generation sequencing. A promising method for pyramiding several stress-tolerance genes and creating rice cultivars with long-lasting resilience to coupled abiotic stresses under shifting climatic conditions is to combine molecular breeding with genome-editing technologies like CRISPR/Cas systems.

There may also be trade-offs associated with QTL pyramiding. A donor allele that improves stress tolerance may be linked to undesirable traits such as delayed maturity, altered plant architecture, grain-quality changes, reduced biomass, or other linkage-drag effects. The heat-tolerant donor Nagina 22, for example, has useful heat-response traits but also possesses undesirable characteristics such as relatively small grains and weak, elongated stems that can increase lodging risk. Consequently, selection should aim to retain the favorable stress allele while recovering the elite recipient background.

The practical value of molecular breeding therefore depends on validation beyond marker presence. Candidate QTLs should be evaluated in the intended recipient background, under the target environmental conditions, at the relevant developmental stage, and using agronomically meaningful traits such as grain yield, yield components, fertility, and yield stability. Multi-environment testing is particularly important because QTL effects can vary with genetic background and environmental severity. For combined heat, drought, and salinity scenarios, future breeding populations should therefore be evaluated directly under compound-stress environments rather than assuming that pyramiding independently validated single-stress loci will reproduce the same effects under stress combinations.

### 7.3. DNA Marker Applications

DNA marker technologies have progressively shifted rice stress breeding from broad polymorphism screening toward sequence-based identification and prediction of favorable alleles. Early approaches such as random amplified polymorphic DNA (RAPD) were useful for demonstrating genetic diversity and developing stress-associated markers, but their historical applications in wheat, cotton, and tomato have limited relevance to a modern rice review focused on integrated adaptation to drought, salinity, and heat [120].

Despite these contributions, RAPD markers have largely been replaced by more reliable and reproducible marker systems such as simple sequence repeats (SSRs), single nucleotide polymorphisms (SNPs), Kompetitive Allele-Specific PCR (KASP) markers, and genotyping-by-sequencing (GBS) platforms. These high-resolution markers enable accurate mapping of quantitative trait loci (QTLs), genome-wide association studies (GWAS), and genomic selection for complex traits, including drought, salinity, and heat tolerance. In rice, SNP-based markers have become the preferred tools for marker-assisted breeding owing to their high abundance, reproducibility, and suitability for high-throughput genotyping. The integration of advanced DNA marker technologies with high-throughput sequencing, genome-wide association studies, genomic prediction, and CRISPR/Cas-based genome editing is transforming modern rice breeding. These complementary approaches facilitate the identification, validation, and deployment of favorable alleles controlling multiple abiotic stress responses, thereby accelerating the development of climate-resilient rice cultivars adapted to increasingly challenging environmental conditions.

### 7.4. Integrated Multi-Omics Strategies

The integration of multiple omics layers genomics, transcriptomics, proteomics, metabolomics, ionomics, interactomics, and phenomics provides a comprehensive understanding of stress responses. This systems-level approach addresses limitations of single-omics studies by capturing the complexity of molecular interactions and regulatory networks [121]. Multi-omics integration enables identification of important biomarkers, regulatory networks, and genetic targets that enhance plant stress resilience. By decoding gene regulation and transcriptional networks, these techniques reveal critical mechanisms underlying stress tolerance [122]. The development of regulatory networks and pathway maps identifies potential targets for improving resistance through genetic engineering or breeding strategies.

Pan-omics approaches comparing core, dispensable, and unique components across accessions assist in identifying novel genes, proteins, and metabolites responsible for stress tolerance. Databases and online repositories now enable storage, analysis, and retrieval of high-throughput data, providing guidelines for harnessing pan-omics potential in sustainable agriculture. Application of artificial intelligence (AI) and machine learning has emerged as a powerful approach for data integration, pattern recognition, and predictive modeling [123]. These computational tools manage large datasets, identify complex relationships, and accelerate discovery of stress tolerance mechanisms.

However, listing omics layers is not itself an integrated analysis. The following concrete rice examples illustrate how at least two omics layers have been combined under heat, drought, salinity, or their combinations, and explain the biological insight gained beyond a single layer, sample and time alignment, causal-network validation, and confirmation of candidate targets.

#### 7.4.1. Transcriptomics and Metabolomics Under Drought, Heat and Salinity

Hai et al. integrated field phenotyping, transcriptomics, metabolomics, and whole-genome resequencing in two contrasting upland rice cultivars (DB46, drought-tolerant; LK23, drought-sensitive). Transcriptomic analyses revealed that DB46 coordinately upregulated pathways related to fatty acid degradation, α-linolenic acid metabolism, and ROS detoxification, supporting efficient energy mobilization and redox homeostasis. Metabolomic profiling confirmed significant enrichment of lipid and secondary metabolism in DB46, including fatty acid biosynthesis, flavonoid biosynthesis, and phenylalanine metabolism [124]. The integrated analysis identified 337 candidate genes associated with lipid metabolism and redox regulation, including lipoxygenase, protein phosphatase 1 L, and PP2C, revealing a lipid–redox regulatory network that underpins drought tolerance and yield stability. The biological insight gained beyond a single layer was the recognition that coordinated lipid remodeling and redox homeostasis, rather than any individual gene or metabolite-distinguish tolerant from sensitive genotypes. Sample and time alignment were achieved by collecting transcriptomic and metabolomic samples from the same leaf tissues at the booting stage, where phenotypic differences were most pronounced. Whole-genome resequencing provided genomic context for causal inference.

Integrated data analysis using clustering based on one-dimensional self-organizing maps identified two profiles highly correlated with HNT sensitivity. The sensitivity profile included genes related to abiotic stress, hormone metabolism, cell wall, signaling, redox state, transcription factors, secondary metabolites, and defense genes. The tolerance profile shared similar functional categories but showed differences in hormone metabolism and transcription factor responses. Metabolites of the two profiles revealed involvement of GABA signaling, linking the TCA cycle status in sensitive cultivars, and myo-inositol as a precursor for inositol phosphates, linking jasmonate signaling to the HNT response specifically in tolerant cultivars. This mechanistic connection between jasmonate signaling and inositol phosphate metabolism would not have been apparent from transcriptomics or metabolomics alone [125].

Under salinity, integrated transcriptomic–ionomic–metabolomic analyses of contrasting rice genotypes have shown that tolerant lines coordinate lower Na^+^ accumulation with higher K^+^ retention and accumulate specific osmolytes and flavonoids. When ionomic data are overlaid on transcriptomic and metabolomic networks, candidate genes such as *OsHKT1;5*, *OsSOS1*, and *OsNHX1* emerge as hubs linking ion homeostasis to osmotic adjustment and antioxidant defense. This integration reveals that salinity tolerance is not simply a function of Na^+^ exclusion but depends on coordinated ion transport, osmolyte accumulation, and redox regulation-an insight that requires at least two omics layers to resolve.

#### 7.4.2. Causal-Network Validation and Candidate Target Confirmation

In the systems breeding framework proposed by Kumar [126], multi-omics-informed GWAS combined with co-localization of eQTLs and protein QTLs in drought-stressed reproductive tissues highlighted high-confidence candidate genes including *OsNAC14*, *OsbZIP23*, and *DRO1* that explain the physiological basis of water-deficit adaptation. A tiered candidate prioritization pipeline is proposed to advance loci supported by convergent genomic, transcriptomic, proteomic, and field-level evidence toward practical breeding deployment. Similarly, in the lipid–redox study, the 337 candidate genes were prioritized based on convergence across transcriptomic, metabolomic, and genomic layers, with lipoxygenase and PP2C identified as high-confidence candidates for functional validation [126].

#### 7.4.3. AI and Machine Learning Applications

AI and ML are valuable for data integration, pattern recognition, and prediction in rice stress biology, but their use must specify task, input data, validation, interpretability, and limitations. Gunnula et al. used a SHAP-optimized stacking ensemble with root anatomical and physiological traits to classify drought tolerance (81.8% accuracy), identifying H_2_O_2_, relative water content, and endodermis inner circumference as key predictors; cross-validation supported interpretability, but destructive root sampling limits breeding application [127]. Lu et al. [128] developed RiceSNP-ABST, a deep-learning model that identifies abiotic stress-associated SNPs from genomic sequence; predictions were experimentally validated, but it requires large annotated datasets and may not generalize to rare or novel stress combinations. Another research shows that an ANN-based multi-target model trained on literature-curated multi-species data to predict stress-combination impacts (76.33% accuracy), with rice yield reductions under combined drought and heat validated; however, accuracy depends on training literature coverage and omits unrepresented genotype × environment effects [129]. A biomarker-gene ML model predicted drought-stress perception in rice accessions with 75% accuracy, but robustness across genetic backgrounds and environments remains limiting. AI/ML are most powerful as task-specific, validated, interpretable complements to experiments. Integrated multi-omics likewise requires sample/time alignment, causal-network validation, and confirmed targets. Progress will come from combining multi-omics, high-throughput phenotyping, GWAS, genomic selection, and multiplex CRISPR/Cas with field validation, microbiome considerations, and AI-assisted analyses grounded in experimental testing.

## 8. Conclusions and Perspectives

Rice is increasingly exposed to simultaneous heat, salinity, and drought stresses, posing a serious threat to global food security under a changing climate. This review highlights that rice adaptation to these stresses is governed by highly interconnected signaling networks involving stress perception, Ca^2+^ signaling, ROS, MAPK cascades, phytohormone crosstalk, and transcriptional regulation. These coordinated pathways regulate physiological and biochemical adaptations, including osmotic adjustment, ion homeostasis, antioxidant defense, molecular chaperone activity, and stress-responsive gene expression, enabling plants to survive under adverse environmental conditions.

Despite significant advancements, several obstacles still exist. Genetic improvement is challenging because abiotic stress tolerance is a complicated quantitative trait regulated by multiple genes with small individual effects, significant epistatic interactions, and powerful environmental influences. Additionally, different developmental phases have different levels of stress sensitivity, with the reproductive phase being especially susceptible. Most significantly, while rice plants in the field are frequently subjected to dynamic and overlapping combinations of heat, drought, and salinity, existing knowledge is primarily drawn from controlled experiments with specific stresses.

Therefore, main stress sensors should be identified, the integration of Ca^2+^, ROS, MAPK, and hormone signaling networks under combined stresses should be clarified, and organelle-specific signaling and epigenetic stress memory should be understood. The identification and validation of important regulators controlling multi-stress tolerance will be accelerated by combining multi-omics technologies with high-throughput phenotyping, genome-wide association studies, genomic selection, and sophisticated genome-editing techniques such as multiplex CRISPR/Cas systems. However, the integration of these approaches must be grounded in concrete experimental design. Sample and time alignment across omics layers, causal-network validation through genetic and biochemical experiments, and functional confirmation of candidate targets are essential requirements for translating multi-omics discoveries into breeding applications. AI and machine learning tools—including SHAP-optimized stacking ensembles for trait classification, deep learning models for SNP prediction, and ANN-based models for combined stress impact prediction—offer powerful complementary approaches, but their predictions must be interpreted with explicit acknowledgment of their dependence on training data, their limited extrapolative capacity, and the need for experimental validation under field conditions. To enhance the prediction of stress-responsive networks, more focus should be given to verifying candidate genes under actual field settings, taking advantage of advantageous plant–microbiome interactions, and integrating AI-assisted multi-omics investigations with rigorous experimental validation.

Ultimately, developing climate-resilient rice cultivars will require an integrated systems biology approach that combines molecular genetics, functional genomics, precision breeding, genome editing, and sustainable crop management. Such multidisciplinary strategies will be essential for improving rice productivity and grain quality under increasingly complex environmental conditions, thereby contributing to global food security and sustainable agricultural development.

## Data Availability

No new data were created or analyzed in this study. Data sharing is not applicable to this article.

## Abbreviations

SOS	salt overly sensitive
MAPK	mitogen-activated protein kinase
ROS	reactive oxygen species
MSL	Mechanosensitive Channel of Small Conductance-Like
MCA	MID1-Complementing Activity
PZO1	PIEZO1
BON	BONZAI
ABA	abscisic acid
CBLs	calcineurin B-like proteins
CIPKs	CBL-interacting protein kinases
CDPKs	calcium-dependent protein kinases
FER	FERONIA
GIPC	glycosylinositol phosphorylceramide
CNGC	cyclic nucleotide-gated channel
HSP	Heat Shock Protein
phyB	Phytochrome B
ATP	Adenosine Triphosphate
BR	brassinosteroids
GA	gibberellins
SA	salicylic acid
SL	strigolactones
CK	cytokinins
MAB	Marker-assisted breeding
HTS	high-throughput sequencing
GWAS	genome-wide association studies
